# Marked differences in CRP genotype frequencies between the Fulani and sympatric ethnic groups in Africa

**DOI:** 10.1186/1475-2875-8-136

**Published:** 2009-06-22

**Authors:** Elisabeth Israelsson, Mattias Ekström, Amre Nasr, Amagana Dolo, Susannah Kearsley, Gishanthi Arambepola, Manijeh Vafa Homann, Bakary Maiga, Ogobara K Doumbo, Gehad ElGhazali, Hayder A Giha, Marita Troye-Blomberg, Klavs Berzins, Per Tornvall

**Affiliations:** 1 Department of Immunology, Wenner-Gren Institute, Stockholm University, Stockholm, Sweden; 2 Department of Cardiology, Karolinska University Hospital Solna, 17176 Stockholm, Sweden; 3 Malaria Research and Training Center (MRTC), Faculty of Medicine and Pharmacy, University of Bamako, Bamako, Mali; 4 Department of Immunology, Faculty of Medicine, King Fahad Medical City, Riyadh, Saudi Arabia; 5 Department of Microbiology and Immunology, Faculty of Medicine, University of Khartoum, Sudan; 6 Malaria Research Center, Department of Biochemistry, Faculty of Medicine, University of Khartoum, Khartoum, Sudan; 7 Department of Medical Biochemistry, College of Medicine and Health Sciences, Arabian Gulf University, Manama, Bahrain

## Abstract

**Background:**

C-reactive protein (CRP) is an acute phase protein that can activate various immune cells and bind to certain Fcγ receptors. The latter may compete with the binding of IgG antibodies to these receptors and could thereby interfere with the antigen-specific immune response. Polymorphisms in the promoter region of the *CRP *gene have been strongly associated with the plasma concentration of CRP. The known lower susceptibility to malaria in the Fulani ethnic group, as compared to their sympatric neighbours in Africa, has been linked to different genetic backgrounds. The present study was performed to investigate if polymorphisms in the CRP gene could contribute to the lower susceptibility to malaria seen in the Fulani ethnic group.

**Methods:**

The CRP -717 T>C, -286 C>T>A, and +1444 C>T polymorphisms were analysed in asymptomatic Fulani and non-Fulani individuals from Mali and Sudan using Pyrosequencing T and TaqMan r MGB probes.

**Results:**

The rare -286 A allele, previously shown to be associated with increased CRP expression and plasma levels, was shown to be more frequent in the non-Fulani ethnic groups as compared to the sympatric Fulani ethnic group both in Mali and Sudan. The common -717 T allele was more prevalent in the non-Fulani ethnic group compared to the sympatric Fulani ethnic group, but only in Mali. The parasite prevalence was increased for the -286 A allele, but not for the -717 T allele. No differences regarding genotype frequency or parasite prevalence were seen for +1444 C>T.

**Conclusion:**

This study indicate that CRP may play an important role in the immune responses to malaria, and that the -286 C/T/A CRP polymorphism may be a contributing factor to the lower susceptibility to malaria seen in the Fulani.

## Background

Malaria, caused by parasites from the *Plasmodium *genus, is a prevalent infectious disease worldwide. The Fulani ethnic group in Africa has shown a lower susceptibility to malaria as compared to their sympatric neighbours, despite being similarly exposed to malaria and having no obvious differences in socio-cultural circumstances. The Fulani have lower parasite rates and parasitaemia, higher anti-malarial immune responses and higher spleen rates than other sympatric groups [1-3]. It has been previously shown that this relative resistance to malaria in the Fulani is pathogen related and not caused by a higher general reactivity of the immune system [4]. A number of studies have reported differences between Fulani and non-Fulani groups in polymorphic immune-related genes [5,6]. Recently, such an inter-ethnic difference in the allele frequencies of a polymorphism in the gene encoding Fca receptor (R) IIa, resulting in an arginine (R)/histidine (H)-131 receptor diversity was demonstrated [7,8].

C-reactive protein (CRP) is an acute phase protein, the levels of which increase rapidly in the circulation during infection and/or inflammation. CRP has the ability to activate various immune cells and has also shown a possible capacity to bind to certain FcγR [9]. Fc receptors are widely expressed on leukocytes and they are important players in the immune response, the binding of antibodies to these receptors leading to activation and onset of many important biological events [10]. Interestingly, CRP shows a sequence homology to the regions in IgG that are important for FcγR binding [9], indicating that the two proteins may bind to a common site on these receptors. The binding of CRP may, therefore, compete with the binding of IgG antibodies to these receptors, and could thereby interfere with the antigen-specific immune response. The FcγRIIa has been shown to have a higher affinity for CRP if the R131 allele of the receptor is present [11], the same allele that in some studies has been related to protection against malaria [12]. Previous studies on falciparum malaria have related high circulating CRP levels with parasite density and severity of the malaria infection [13-15]. Interestingly, in individuals having an asymptomatic *Plasmodium falciparum *infection, the plasma levels of CRP are low [16,17], suggesting that the increased CRP levels found in *P. falciparum *symptomatic individuals are not merely an acute phase response. The role of CRP in *P. falciparum *infections is not clear. CRP has been associated with complement mediated haemolysis of infected erythrocytes and subsequent anaemia [18], but CRP has also been implicated in the defence against pre-erythrocytic stages of malaria [19]. Importantly, CRP induces the anti-inflammatory interleukin (IL)-10 [20], which could affect the early immune response seen in malaria infections. The influence of genetic variations on the levels of circulating CRP is estimated to be 40–60% [21], and single nucleotide polymorphisms (SNPs) in different cytokine genes [22,23] and the *CRP *gene [24] have been suggested to be associated with the circulating levels of CRP. The strongest association with the circulating concentrations of CRP has been shown for the tri-allelic -286 SNP [25,26], and it has been suggested that this is a truly functional polymorphism in the CRP gene [24]. Previous studies of the -286 C>T>A CRP SNP have revealed that the A allele is more common in African American than in Caucasian populations, and that this difference in genotypes is associated with higher CRP levels in the former population [27]. This suggests that CRP could have been selected for in the African ancestors, as a factor beneficial for survival from infectious diseases.

In order to investigate the possible involvement of CRP in innate immunity against malaria, and to further define potential immunological factors contributing to the ethnic differences in malaria susceptibility, the -717 T>C (rs2794521), -286 C>T>A (rs3091244,) +1444 C>T (rs1130864) CRP genotypes and malariometric indices were analysed in individuals of sympatric ethnic groups from two countries, Mali and Sudan, with markedly different malaria endemicity.

## Methods

### Study areas

The study area in Mali is located in the Mopti area about 850 km Northeast of Bamako, the capital of Mali. Four villages, Mantéourou, Naye, Binédama, and Anakédié were identified for the study. Malaria transmission is mesoendemic in the area, with *P. falciparum *as the main parasite species. The entomological inoculation rate is similar in both ethnic groups [3]. In this area, the dry season extends from October to May and the rainy season from July to October.

The study area in Sudan is located in the Daraweesh village in the Gedaref State in eastern Sudan, 450 km from Khartoum and 16 km from the Gedaref town. Malaria transmission is markedly seasonal and unstable, and annual peak parasite prevalence ranges from 1 to 40% in different years, and *P. falciparum *is responsible for >96% of the malaria cases [28].

### Human samples

In Mali, blood was collected during the rainy season in September 2005 from 166 Fulani (age range 1–60 years, median age 15 years, 51% males) and 608 non-Fulani represented by the Dogon (98%) and Rimaibé (2%) ethnic groups (age range 7 month – 61 years, median age 8 years, 44% males). All individuals were asymptomatic at the time of collection, and parasite prevalence was detected by microscopy. For PCR, finger prick blood was collected on filter papers. Spleen enlargement was assessed by palpation and graded according to the Hackett score (0–5). The proportion of individuals that had enlarged spleens was denoted spleen rate. Informed consent was obtained from all participants or their guardians and the Ethical committee of the Faculty of Medicine and Pharmacy, University of Bamako, in Mali and the National Ethics Committee in Sweden approved the study.

In Sudan, blood was collected, as part of a longitudinal study, in May before the rainy seasons between 2004 and 2006, from 225 Fulani (age range 4–75 years, median age 15 years, 36% males) and 100 non-Fulani represented by the Four (22%), Hausa (28%) and Masaleit (50%) ethnic groups (age range 2–55 years, median age 17 years, 41% males). All the individuals were asymptomatic and parasite negative by microscopy at the time of collection. Three ml of peripheral blood were collected from all individuals into vacuum EDTA tubes. The study received ethical approval from the ethical Committee of University of Khartoum and national clearance from the Sudanese Ministry of Health.

### DNA preparation

DNA was extracted from filter papers and venous blood with Chelex-100 as described previously [5], and then stored at -20°C. In brief, discs from filter paper or 25 μl from peripheral blood were incubated overnight in 1 ml of 0.5% saponin in PBS at 4°C, and were then washed 15–30 minutes in 1 ml PBS at 4°C. The discs or the pellets, were then boiled in 200 μl of 5% Chelex-100 in water for 15 minutes, and subsequently DNA was collected in the supernatants after centrifugation at 8,000 × g for three minutes.

### CRP genotyping

The -286 C>T>A CRP polymorphism (rs3091244) was analysed using the pyrosequencing method. PCR primers were designed to amplify the -*286 C>T>A *polymorphism, using the forward primer 5'-TGT TTT CCT CAT TTC CCA GTC T-3' and the reverse primer 5'-biotinylated TGG CTA TCT ATC CTG CGA AAA T-3' (MWG-Biotech AG, Ebersberg, Germany). The PCR amplification was performed in a 40 μl reaction using 8 μl of genomic DNA template, 20 μl iProof *T *High-Fidelity Master Mix (BIO-RAD Laboratories, Hercules, CA), and 1.5 μl of each primer (10 pmol/μl). The PCR was carried out in an Eppendorf Mastercyclerr (Eppendorf AG, Hamburg, Germany) using a 30 sec denaturation at 98°C, followed by 35 cycles with 98°C for 10 sec, 59°C for 20 sec and 72°C for 15 sec. The final extension was at 72°C for 5 min. The sequencing primer was 5'-GTG CAC CCA GAT GGC-3' (MWG-Biotech AG), and the nucleotide dispensation order was GCA CGT ACA GT. Sequencing was carried using the PSQ™ 96MA pyrosequencing apparatus (Pyrosequencing, Biotage AB, Uppsala, Sweden) according to the instructions from the manufacturer.

The -717 T>C (rs2794521) and +1444 C>T (rs1130864) CRP polymorphisms were analysed with Taqmanr MGB Probes from Applied Biosystems according to the manufacturers protocol.

### Statistical analysis

All statistical analyses were performed in StatView version 5.0.1 unless stated otherwise. Differences in genotype frequencies and allele frequencies between the ethnic groups were tested for statistical significances using χ^2 ^tests. Analyses of the estimated haplotype frequencies and associations of alleles were performed in Unphased (version 2.403) [29]. Linkage analysis was performed in FStat version 2.9.3.2. A P-value of 0.05 or less was considered as statistically significant.

## Results

### CRP genotype and allele frequencies

The genotype frequency of the -286 C>T>A polymorphism was similar in both countries (Table 1) and was found to be in Hardy-Weinberg equilibrium. The allele frequencies were significantly different between the two ethnic groups in Mali and Sudan (P < 0.0001) (Table 1), the A allele being at significantly higher frequency in the non-Fulani groups than in the Fulani groups (Mali: OR = 2.79, P = 1.4 × 10^-8 ^and Sudan: OR = 2.51, P = 8.2 × 10^-7^, respectively). While the C allele was found to be at a lower frequency in the Malian non-Fulani as compared to the Fulani (OR = 0.36, P = 3 × 10^-10^), the T allele frequency was lower in the Sudanese non-Fulani group (OR = 0.28, P = 4 × 10^-7^).

**Table 1:** Genotype and allele frequencies of the -286 CRP C>T>A polymorphism (rs3091244) in the sympatric ethnic groups in Mali and Sudan

	*Mali*		*Sudan*	
	*Fulani (n = 166)*	*Non-Fulani (n = 608)*	*Fulani (n = 225)*	*Non-Fulani (n = 100)*
**Genotype frequency**				
				
*AA*	6 (4%)	107 (18%)	11 (5%)	17 (17%)
*AC*	37 (22%)	170 (28%)	42 (19%)	39 (39%)
*AT*	12 (7%)	113 (19%)	29 (13%)	6 (6%)
*CC*	62 (29%)	80 (13%)	65 (29%)	25 (25%)
*CT*	41 (25%)	98 (16%)	56 (25%)	12 (12%)
*TT*	8 (5%)	40 (7%)	22 (10%)	1 (1%)
*P-value*	< 0.0001		< 0.0001	
				
**Allele frequency**				
				
*A*	0.18	0.39	0.21	0.39
*C*	0.61	0.36	0.51	0.51
*T*	0.21	0.25	0.28	0.10
*P-value*	< 0.0001		< 0.0001	
				
**Frequency of A-allele carriers**^a^				
				
*A*	55 (33%)	390 (64%)	82 (36%)	62 (62%)
*Non-A*	111 (67%)	218 (35%)	143 (64%)	38 (38%)
*P-value*	< 0.0001		< 0.0001	

^a ^Frequency of individuals with or without at least one A-allele of the -286 CRP polymorphism

Further, the possible influence of the A allele on some malariometric data collected for the Mali study group were analysed, and the results revealed that individuals being parasite positive were more likely to be A-allele carriers than parasite negative individuals (p = 0.04) (Table 2), but no differences were seen for genotypes or when separated based on ethnicity. No differences were seen in the frequency of splenomegaly (A-allele carriers with enlarged spleen: 25%, non A-allele carriers with enlarged spleen: 21%, p = 0.2) or haemoglobin levels (median (range): A-allele carriers: 11 (5.4–17.5), non A-allele carriers: 11 (3.3–17.7), p = 0.8) between A- and non-A allele carriers.

**Table 2:** Frequencies of *P. falciparum *parasite positive and negative individuals in the -286 CRP genotypes and A-allele carriers from Mali.

	All			Fulani			Non-Fulani		
Parasites^a^:	n	Positive	Nagative	n	Positive	Nagative	n	Positive	Nagative
**CRP genotype**									
									
AA	113	0.16	0.14	6	0.06	0.03	107	0.18	0.17
AC	207	0.31	0.25	37	0.22	0.22	170	0.32	0.26
AT	125	0.17	0.16	12	0.13	0.06	113	0.18	0.19
CC	142	0.12	0.21	62	0.31	0.39	80	0.09	0.15
CT	139	0.18	0.18	41	0.25	0.25	98	0.16	0.16
TT	48	0.06	0.06	8	0.03	0.05	40	0.07	0.07
P-value	0.2			0.7			0.4		
									
**A-allele carrier**									
									
A-allele	445	0.64	0.55	55	0.41	0.31	390	0.68	0.62
Non-A	329	0.36	0.45	111	0.59	0.69	218	0.32	0.38
P-value	0.04			0.3			0.18		

^a ^Parasite status detected by microscopy.

The genotype frequency of the -717 T>C polymorphism was similar in both countries (Table 3), and was found to be in Hardy-Weinberg equilibrium. Both the genotype and the allele frequencies of the -717 T>C polymorphism showed a significant difference in Mali, but not in Sudan. No differences in parasite positive individuals, splenomegaly or haemoglobin levels were found between the different genotypes (data not shown).

**Table 3:** Genotype and allele frequencies of the -717 CRP T>C polymorphism (rs2794521) in the sympatric ethnic groups in Mali and Sudan

	*Mali*		*Sudan*	
	*Fulani (n = 166)*	*Non-Fulani (n=165)*	*Fulani (n = 211)*	*Non-Fulani (n = 45)*
**Genotype frequency**				
				
*CC*	8 (5%)	2 (1%)	12 (6%)	3 (7%)
*CT*	48 (29%)	21 (13%)	59 (28%)	9 (20%)
*TT*	110 (66%)	142 (86%)	140 (66%)	33 (73%)
*P-value*	< 0.0001		0.5	
				
**Allele frequency**				
				
*C*	0.19	0.08	0.20	0.17
*T*	0.81	0.92	0.80	0.83
*P-value*	< 0.0001		0.5	
				
**Frequency of T-allele carriers **^a^				
				
*T*	158 (95%)	163 (99%)	12 (6%)	199 (94%)
*Non-T*	8 (5%)	2 (1%)	3 (7%)	42 (93%)
*P-value*	0.06		0.8	

^a ^Frequency of individuals with or without at least one T-allele of the -717 CRP polymorphism

The genotype frequency of the +1444 C>T polymorphism was similar in both countries, and was found to be in Hardy-Weinberg equilibrium. No differences in either genotype, allele frequencies (Table 4) or malariometric indexes (data not shown) in neither Mali nor Sudan were detected for the +1444 C>T CRP polymorphism

**Table 4:** Genotype and allele frequencies of the +1444 CRP C>T polymorphism (rs1130864) in the sympatric ethnic groups in Mali and Sudan

	*Mali*		*Sudan*	
	*Fulani (n = 166)*	*Non-Fulani (n = 165)*	*Fulani (n = 213)*	*Non-Fulani (n = 46)*
**Genotype frequency**				
				
*CC*	123 (74%)	130 (79%)	160 (75%)	32 (70%)
*CT*	41 (25%)	34 (20%)	47 (22%)	14 (30%)
*TT*	2 (1%)	1 (1%)	6 (3%)	0 (0%)
*P-value*	0.5		0.3	
				
**Allele frequency**				
				
*C*	0.86	0.89	0.86	0.85
*T*	0.14	0.11	0.14	0.15
*P-value*	0.3		0.7	
				
**Frequency of T-allele carriers **^a^				
				
*T*	43 (26%)	35 (21%)	53 (25%)	14 (30%)
*Non-T*	123 (74%)	130 (79%)	160 (75%)	32 (70%)
*P-value*	0.3		0.4	

^a ^Frequency of individuals with or without at least one T-allele of the +1444 CRP polymorphism

### Haplotype associations between CRP -717 T/C, -286 C/T/A, +1444C/T and FcγRIIa 131 R/H

Both the *CRP *and *FcγRIIa *genes are located on chromosome 1q21–1q23. Previous investigations of the two allotypes of the FcγR131H (rs1801274) polymorphism showed a difference in allele frequency between the Fulani and non-Fulani groups in both Mali [7] and Sudan [30]. These findings, together with the results in this study regarding the -286 CRP polymorphism, led us to investigate if the haplotype patterns in the FcγRIIa R131H and CRP -286, -717 and +1444 polymorphisms differ between the different ethnic groups. The polymorphisms were not in linkage disequilibrium. A clear pattern was seen with regards to haplotype patterns, haplotypes containing the A allele of the -286 CRP polymorphism being exclusively associated with the non-Fulani group in both Mali and Sudan (Table 5). This association was regardless of the FcγRIIa allotypes or the -717 and +1444 CRP polymorphisms.

**Table 5:** Haplotype frequencies of the FcγRIIa 131 R/H*, CRP -286 C/T/A, CRP -717 C/T and CRP +1444 T/C

**Mali**												
		FcγRIIa131R/H CRP-286C/T/A		CRP-286C/T/A CRP-717C/T	CRP-286C/T/A CRP+1444T/C	FcγRIIa131R/H CRP-286C/T/A CRP-717C/T		CRP-286C/T/A CRP-717C/T CRP+1444T/C	FcγRIIa131R/H CRP-286C/T/A CRP+1444T/C		FcγRIIa131R/H CRP-286C/T/A CRP-717C/T CRP+1444T/C	
*Reference haplotype*		*HA*		*AC*	*AC*	HAC		*ACC*	*HAC*		*HACC*	

	N	HA	RA	AT	AC	HAT	RAT	ATC	HAC		HATC	RATC
*Non-Fulani*	159	0.20	0.19	0.39	0.39	0.20	0.19	0.39	0.21		0.20	0.19
*Fulani*	164	0.09	0.09	0.18	0.18	0.09	0.09	0.18	0.7		0.09	0.09
*OR*		2.57	2.26	2.79	2.79	2.58	2.29	2.79	3.6		2.6	2.28
*P-value*		0.0001	0.0006	1 × 10^-8^	1 × 10^-8^	0.0001	0.0005	1 × 10^-8^	1 × 10^-8^		0.0001	0.0004

**Sudan**												

	N	HA	RA	AT	AC	HAT	RAT	ATC	HAT	RAC	HATC	RATC
*Non-Fulani*	100	0.21	0.18	0.41	0.41	0.22	0.18	0.41	0.25	0.17	0.24	0.17
*Fulani*	225	0.13	0.08	0.21	0.21	0.13	0.08	0.21	0.12	0.08	0.13	0.09
*OR*		1.88	2.52	2.65	2.66	2.05	2.6	2.65	2.33	2.24	2.19	2.42
*P-value*		0.007	0.0003	0.0001	0.0001	0.03	0.006	0.0001	0.005	0.03	0.01	0.02

* The data for the FcγRIIa 131 R/H from Mali was obtained from Israelsson *et al *[7], and from Sudan from Nasr *et al *[30]

## Discussion

The present study demonstrates striking differences regarding CRP single nucleotide polymorphisms between sympatric ethnic groups, which may contribute to the differences in their susceptibility to malaria. In particular, the CRP SNP -286 C/T/A showed a marked difference in genotype frequencies between Fulani and non-Fulani individuals in two independent cohorts from Mali and Sudan. Lower frequencies of the A-allele of this SNP, previously associated with higher circulating CRP concentrations, were demonstrated in the two independent Fulani groups as compared to other sympatric ethnic groups. Moreover, a higher parasite prevalence was detected in individuals with the A allele, supporting the cause-relationship. The other two analysed polymorphisms did not show any consistent differences between Mali and Sudan, and in the haplotype analysis it was clearly the -286 A-allele that showed a consistent association with one ethnic group in both countries. The results suggest that the -286 CRP polymorphism may be a contributing factor in the lower susceptibility to malaria seen in the Fulani group.

However, the hypothesis that the -286 A-allele is associated with protection against infectious diseases due to a selection in African populations was refuted. The reason for this is not clear, but the A-allele might be associated with a lower risk of other diseases, as discussed below.

So far, inconclusive results, suggesting both a beneficial and harmful role of CRP in malaria [18,19], have made it difficult to conclude about its role in the disease. However, a recent study showed that binding of CRP to infected red blood cells (RBCs) increased the removal of damaged RBCs from the circulation [31], which could lead to a more pronounced anaemia. Further, the control of *P. falciparum *parasitaemia is dependent on a pro-inflammatory response, and an uncontrolled inflammation is suggested to cause severe symptoms [32]. Higher levels of circulating CRP could influence the IL-10 levels and thereby affect the delicate balance between pro- and anti-inflammatory responses, leading to a reduced control of parasitaemia. Support for this notion comes from previous studies showing high circulating CRP levels in individuals with high parasitaemia [13,14], indicating an effect of CRP on parasite clearance. IL-10 is also involved in the generation of peripheral regulatory T cells (Treg) [33]. The up-regulation of Tregs in a malaria infection has been shown to increase the parasite growth [34], and a recent study demonstrated that the Fulani present a functionally impaired Treg repertoire as compared to their sympatric neighbours [35]. Moreover, CRP may show direct effects on dendritic cell differentiation, maturation and function [36], and neutrophil chemotaxis and signalling [37]. Since dendritic cells are important in initiating and regulating immune responses, and neutrophils have shown a protective effect against malaria *in vitro *[38,39], such influences may seriously hamper an effective immune response.

Low levels of CRP have been shown for a number of autoimmune diseases [40], and with the predicted low levels of circulating CRP in the Fulani groups, it could be assumed that the Fulani may be more susceptible to autoimmune diseases, and this is true for some disorders, e.g. diabetes [41]. The results of the present study may suggest, that a high level of circulating CRP can be detrimental for malaria protection, although by a yet unknown mechanism. The supporting promising finding of a higher frequency of parasite positive individuals among -286 A-allele carriers than among the non-A-allele carriers further strengthens this hypothesis. One limitation of the present study is that no reliable data on plasma levels of CRP could be presented due to lack of plasma in Mali and to lack of standardisation of blood sampling in Sudan. In future studies there is a need to study both unstimulated and plasma levels stimulated by malaria in relation to CRP genotypes in Africa.

The FcγRIIa 131 H/R polymorphism has been associated with malaria susceptibility in several studies [12,42,43]. CRP binds with a higher affinity to the receptor expressed by the R allele [11], which might have a competitive effect on the binding of the previously shown malaria protective immunoglobulins (Ig) G1 and IgG3 [44], thereby interfering with the protection against malaria. Since the *CRP *gene and the *FcγRIIa *gene are located in the same region on chromosome 1, the possibility of a susceptibility locus on this chromosome was investigated. Although the linkage analysis did not show any linkage between these two polymorphisms, associations were found between haplotypes containing the -286 A-allele, regardless of the FcγRIIa allotype or the -717 or +1444 CRP polymorphisms, and the non-Fulani groups. This finding suggests that the -286 CRP polymorphism could have a stronger impact on malaria susceptibility than the other investigated variants. The finding that there are more parasite positive individuals among the A-allele carriers than among the non-A allele carriers, further strengthens this suggestion. The sample size in this study was probably too small for making any firm conclusions, but the results strongly indicate a need for further studies on the impact of CRP in relation to susceptibility to malaria.

## Conclusion

In conclusion, this study has demonstrated a marked difference in CRP genotype frequencies in two independent samples of Africans with low susceptibility to malaria as compared to sympatric ethnic groups. The -286 CRP polymorphism that was analysed has been shown to be functional *in vitro*, and associated with *in vivo *CRP levels in the circulation. This study further shows that the high producing -286 A-allele may be associated with parasitaemia, and it can, therefore, be speculated that CRP play a role in the early immune response to malaria infection. This may possibly occur by inhibiting dendritic cells and/or neutrophils, by competition with the binding of malaria specific IgG antibodies to Fcγ receptors, by enhance the clearance of infected RBCs, leading to more severe anaemia, or a deviation towards an anti-inflammatory cytokine response induced by CRP. Further research on the effect of CRP on malaria susceptibility is warranted, since recent studies on new therapeutic agents, that lower the circulating CRP levels [45], might open up new adjunct treatment options against malaria.

## Competing interests

The authors declare that they have no competing interests.

## Authors' contributions

EI participated in the design and conception of the study, collection of samples, performed the CRP genotypings and the CRP measurements, carried out the statistical analyses and drafted the manuscript. ME participated in the design and conception of the study and helped to draft the manuscript. AN was responsible for design of sample collection in Sudan and helped to draft the manuscript. AD was responsible for design of sample collection in Mali and helped to draft the manuscript. SK helped in the design of TaqMan analysies and performed the CRP genotyping and helped to draft the manuscript. GA participated in the Pyrosequencing analysis and the CRP measurements and helped to draft the manuscript. MV helped in the collection of samples and helped to draft the manuscript. BM examined all participants from Mali and participated in design and collection of samples and helped to draft the manuscript. OKD was responsible for design of sample collection in Mali and helped to draft the manuscript. GE was responsible for design of sample collection in Sudan and helped to draft the manuscript. HAG was responsible for design of sample collection in Sudan and helped to draft the manuscript. MTB participated in the design and the conception of the study and helped to draft the manuscript. KB participated in the design and the conception of the study and drafted the manuscript. PT participated in the design and the conception of the study, performed the CRP genotyping and helped to draft the manuscript. All authors read and approved the final manuscript
